# First evidence for temperature’s influence on the enrichment, assembly, and activity of polyhydroxyalkanoate-synthesizing mixed microbial communities

**DOI:** 10.3389/fsysb.2024.1375472

**Published:** 2024-08-14

**Authors:** Anna Trego, Tania Palmeiro-Sánchez, Alison Graham, Umer Zeeshan Ijaz, Vincent O’Flaherty

**Affiliations:** ^1^ Microbial Ecology Laboratory, Microbiology, School of Biological and Chemical Sciences, College of Science and Engineering, University of Galway, Galway, Ireland; ^2^ Water Engineering Group, School of Engineering, The University of Glasgow, Glasgow, United Kingdom

**Keywords:** polyhydroxyalkanoates, mixed communities, microbiome, bioplastics, temperature

## Abstract

Polyhydroxyalkanoates (PHA) are popular biopolymers due to their potential use as biodegradable thermoplastics. In this study, three aerobic sequencing batch reactors were operated identically except for their temperatures, which were set at 15 °C, 35 °C, and 48 °C. The reactors were subjected to a feast–famine feeding regime, where carbon sources are supplied intermittently, to enrich PHA-accumulating microbial consortia. The biomass was sampled for 16S rRNA gene amplicon sequencing of both DNA (during the enrichment phase) and cDNA (during the enrichment and accumulation phases). All temperatures yielded highly enriched PHA-accumulating consortia. Thermophilic communities were significantly less diverse than those at low or mesophilic temperatures. In particular, *Thauera* was highly adaptable, abundant, and active at all temperatures. Low temperatures resulted in reduced PHA production rates and yields. Analysis of the microbial community revealed a collapse of community diversity during low-temperature PHA accumulation, suggesting that the substrate dosing strategy was unsuccessful at low temperatures. This points to future possibilities for optimizing low-temperature PHA accumulation.

## Introduction

Polyhydroxyalkanoates (PHAs) are a group of biopolymers which have garnered increased attention due to their biodegradability and thermoplastic properties. They are potential substitutes for conventional petroleum-based plastics, significantly reducing the environmental impacts associated with fossil fuel consumption and global plastic pollution (Palmeiro-Sánchez et al., 2022). They are notably the only known class of biopolymers with thermoplastic properties. PHAs are produced within a cell’s cytoplasm as “intracellular inclusions”, or “granules” (Serafim et al., 2008). A cell will accumulate PHAs under stress induced by nutrient limitations, and it can use them later as a carbon or energy source (Carvalho et al., 2014).

Industrial PHA production harnesses this capacity in order to maximize PHA production by employing a strategic feeding regime generally referred to as “feast–famine” (Van Loosdrecht et al., 1997). This has been shown to successfully enrich for PHA-accumulating organisms by providing a carbon-rich substrate during the feast phase, followed by a prolonged famine period with no carbon additions (Johnson et al., 2010a; Jiang et al., 2011a; Moralejo-Gárate et al., 2011; Palmeiro-Sánchez et al., 2022).

Pure cultures of known PHA-accumulating organisms have been traditionally used for PHA production, and more than 300 microorganisms with PHA cycling capabilities have now been identified (Guzik et al., 2020). Other options to reduce the costs of PHA production currently include the use of pure culture extremophiles, recombinant bacteria, and algae/transgenic plants (Angra et al., 2023). These options, however, often still require the use of a pure substrate, which accounts for roughly 50% of production cost (Zytner et al., 2023). However, mixed cultures were proposed in the late 1990s as a more economical alternative to mitigate the costs associated with sterile cultivation conditions and refined feedstocks (Van Loosdrecht et al., 1997). Mixed culture PHA production includes the added advantages of (i) utilizing wastewater as a feedstock and (ii) process manipulation in terms of polymer composition and purity (Guzik et al., 2020). However, there have been a number of limitations which research has been keen to address. A primary drawback, compared to pure culture PHA production, is the efficiency of the process—or volumetric productivity (Palmeiro-Sánchez et al., 2022). To this end, researchers have identified a number of factors which influence PHA production and likely also the composition and activity of PHA-accumulating microbial communities (although the community dynamics are not always measured). Several examples of such parameters include substrate composition, organic loading rates, pH, C:N ratio, aeration, and temperature (Palmeiro-Sánchez et al., 2023). Recent research has focused on substrate choice and ratio, finding that mixed microbial communities may have a preference for longer (butyric and valeric) rather than shorter acids, and that the ratio of these acids influences production yields (Clagnan and Adani, 2023; Bravo-Porras et al., 2024). Moreover, salinity and feeding mode have also been shown to influence PHA production (Wen et al., 2024).

Temperature, however, is also key to the process and an important cost consideration when scaling up. Operating at lower temperatures could reduce operating costs associated with heating, but operating at higher temperatures could result in significantly higher yields (Estévez-Alonso et al., 2021). To date, the effects of temperature on the PHA production process have been addressed by only a limited number of studies, which have reported operating temperatures between 15 °C and 35 °C (Krishna and Van Loosdrecht, 1999; Johnson et al., 2010b; Jiang et al., 2011a; De Grazia et al., 2017; Inoue et al., 2018). Moreover, the effects of temperature on the enrichment and activity of PHA-accumulating mixed microbial communities is even rarer. Here, for the first time, we report on the structure (DNA) and activity (cDNA) of PHA-accumulating communities enriched at three different temperatures: 15 °C, 35 °C, and 48 °C. Our aims were to: (i) determine how temperature influences the microbiome structure and diversity of enrichments in terms of PHA synthesis potential; (ii) assess the active fraction of the microbial community during PHA accumulation at the three temperatures; (iii) link the microbial community dynamics to the process, providing a holistic view and potential recommendations for process optimization.

## Materials and methods

### System design and operation

**FIGURE 1:**
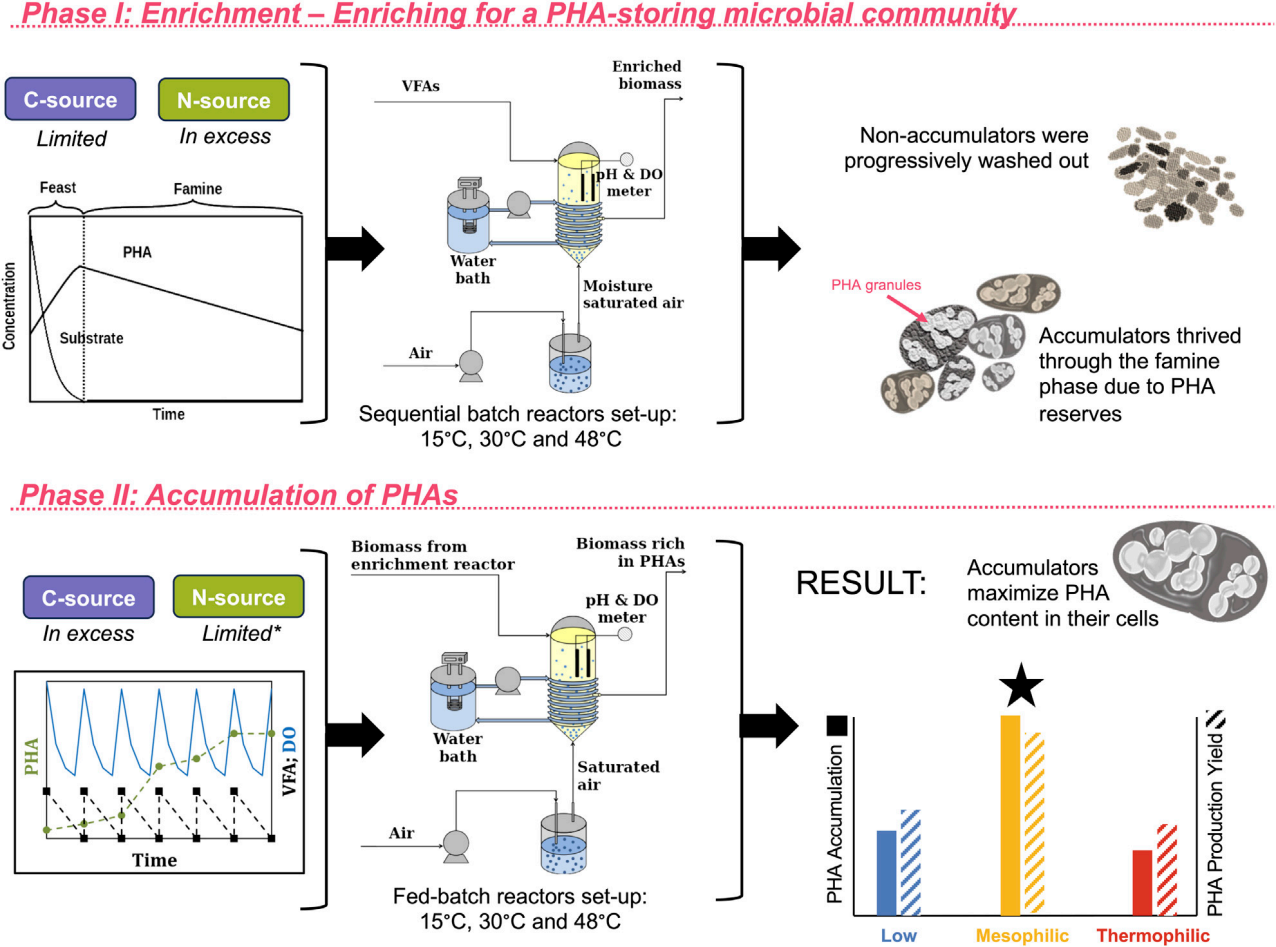
Detailed diagram of the process configuration. During Phase I of the experiment, the enrichment phase, the biomass was enriched for PHA-accumulating organisms. This was achieved by operating the sequencing batch reactors under a feast–famine feeding regime. During the feast phase, the substrate was consumed and PHA-accumulating organisms produced and stored PHA. During the famine phase, when substrate was not supplied, the PHA-accumulating organisms were able to survive on their PHA reserves, while non-accumulators were progressively washed out. During Phase II, the accumulation phase, where the carbon source was not limited and PHA accumulators were allowed to maximize the amount of PHA stored within their cells, PHA accumulation and production yields were highest in the mesophilic system, followed by the low-temperature system, and finally the thermophilic system. Further details on yields and PHA characterization have been previously reported.

The aim was to enrich different mixed microbial cultures able to accumulate PHAs at 15 °C, 30 °C, and 48 °C to compare their microbial composition and accumulation performance. The reactors were kept under the same operational conditions, except for the operating temperature, to observe how this parameter shaped the microbiome (Figure 1). Consequently, three identical aerobic sequential batch reactors (SBRs) were operated in parallel for 332 days under non-sterile conditions.

The SBRs had a working volume of 1.8 L, and temperatures were regulated using a water back jacketed system. All reactors were inoculated with biomass inoculum from the aerobic treatment of industrial dairy effluents (Kilconnell, Ireland). The experiment proceeded in two primary stages: (i) an initial “enrichment” phase, where the biomass inoculum was allowed to develop into a PHA-accumulating consortium by introducing some stressors like the limitation of C-source following a feast–famine sequence; (ii) an “accumulation” phase, where the C-source was not limited and the PHA is maximized within the microbial cells (Figure 1). The substrate was a mixture of volatile fatty acids (VFAs) (2.4 g/L sodium acetate, 0.58 g/L sodium propionate and 0.31 mL/L butyric acid), with an organic loading rate (OLR) of 90 Cmmol/(L d). The solid retention time (SRT) and hydraulic retention time (HRT) were both maintained at 1 day. The SBR cycles were 12 h. A feast–famine feeding regime was applied as a selective pressure for the selection of PHA-accumulating organisms. With this method, the carbon source was limited in each cycle during the famine phase and provided in excess during the feast phase. The reactors were fully aerated, and the dissolved oxygen was measured with a probe to monitor the feast–famine sequences and also to check that there was no oxygen limitation in any of the stages. The pH, NH_4_^+^, VFAs, total solids (TS), and volatile solids (VS) were measured frequently to monitor the continuous operation of each reactor.

In Phase II, accumulation-fed batch reactors were run to maximize the PHA content inside the cells. The jacketed glass reactors had a working volume of 1.8 L and were kept at the same temperature as the enrichment reactors: 15 °C, 30 °C, and 48 °C. The reactors were completely aerated and fully stirred. The inoculum for the accumulation reactors was the biomass of the enrichment SBRs. The substrate was the same VFA mixture as the enrichment but was added in pulses every time the dissolved oxygen concentration increased in the liquid media. No nutrients were added to avoid cell growth; instead, all organic matter was diverted to PHA storage. Again, the pH, NH_4_^+^, TS, and VS were measured every few hours to monitor the continuous operation of each batch reactor fed.

See Palmeiro-Sánchez et al. (2023) for more detailed information about the operational parameters, system performance, and biopolymer properties.

### Analytical methods

TS and VS were analyzed according to the *Standard Methods for the Examination of Water and Wastewater* (APHA, 2005). The PHA content was estimated by gas chromatography (GC) following Smolders et al. (1994). The NH_4_^+^ concentration together with the pH were determined photometrically using a discrete analyzer (Thermo Scientific, US).

### DNA/RNA co-extraction and cDNA synthesis

Nucleic acids were extracted from flocculant biomass (n = 3). In the first part, DNA samples correspond to the inoculum and four consecutive timepoints per temperature during the initial enrichment phase of the trial (n = 39 DNA samples). These samples are referred to as “Inoculum,” “Low_1,” “Low_2,” “Low_3,” and “Low_4,” with similar naming for samples from the mesophilic (“Meso”) and thermophilic (“Thermo”) reactors. In the second part, RNA was isolated from samples (n = 3) corresponding to the enrichment (at two timepoints) and accumulation (one timepoint) phases (n = 27 RNA samples). These samples are referred to as “Enr_Low_1,” “Enr_Low_2,” and “Acc_Low,” where “Enr” denotes the enrichment phase and “Acc” denotes the accumulation phase. Samples from the mesophilic (“Meso”) and thermophilic (“Thermo”) reactors are named similarly. These samples were chosen to compare the active community in the enrichment phase (at two different timepoints) to the active community in the accumulation phase.

For each sample, nucleic acids from 0.1 g of wet biomass were extracted following McAteer et al. (2020) and Trego et al. (2021b). In brief, this method utilizes bead-beating in 1% (w/v) cetyl-trimethylammonium bromide (CTAB; Sigma-Aldrich) buffer, followed by a phenol-chloroform co-extraction. Purified nucleic acids were resuspended in nuclease-free water. Sample concentrations were determined using a Qubit fluorometer (Invitrogen, Carlsbad, CA, USA) while sample quality was assessed using a NanoDrop™ spectrophotometer (Thermo Fisher Scientific, Waltham, MA, USA). DNA was stored at −20 °C, and RNA was stored at −80 °C.

For RNA samples from the enrichment and accumulation phases, cDNA was then synthesized in two steps described by Trego et al. (2020b). In the first step, the samples were DNase treated (to remove any co-extracted DNA) using a TurboDNase kit (AMBION–Invitrogen, Carlsbad, CA, USA). Once gel electrophoresis confirmed the absence of any residual DNA, cDNA was synthesized from the remaining RNA using the Superscript IV (SSIV) Reverse Transcriptase Kit (Thermo Fisher, Waltham, MA, USA). cDNA was then stored at −20 °C.

### High-throughput sequencing and data availability

Nucleic acids from all samples were normalized to 5 ng μL^−1^. Amplification of the V4 region of the 16S rRNA gene was performed on the Illumina MiSeq platform by FISABIO (Valencia, Spain) using the universal bacterial and archaeal primer set 515F and 806R (Caporaso et al., 2011). The sequencing data from this study are available through the NCBI database under the project accession number PRJNA907847 with sample names and SRR numbers (Supplementary Data Sheet S1).

### Bioinformatics and statistical analysis

Abundance tables were generated by constructing amplicon sequencing variants (ASVs) using the Qiime2 workflow with DADA2 (Kozich et al., 2013). Full details are provided at https://github.com/umerijaz/tutorials/blob/master/qiime2_tutorial.md and are similar to methods (bioinformatics and statistics) we previously published (Trego et al., 2021a; Trego et al., 2022). A total of 5,968 ASVs from n = 66 (39 DNA; 27 cDNA) samples were identified. ASVs were classified using the SILVA SSU Ref NR database release v.138. A rooted phylogenetic tree was generated through Qiime2, and a final BIOM file was generated which combined abundance information with taxonomy. Additionally, we used PICRUSt2 (Douglas et al., 2020) within the QIIME environment to recover KEGG enzymes and MetaCyc pathway predictions.

Statistical analysis was performed in R (v.4.2.1) using the combined data generated from the bioinformatics as well as meta-data associated with the study. As a pre-processing step, we removed typical contaminants such as mitochondria and chloroplasts, as well as any ASVs that were unassigned at all levels. This gave a final 66 × 5,607 abundance table, with summary statistics of read distributions for samples as [Min: 11,086; 1st Quartile: 23,324; Median: 26,956; Mean: 48,309; 3rd Quartile: 82,401; Maximum: 124,013].

The Vegan package (Oksanen et al., 2015) was used for alpha and beta diversity analyses (Trego et al., 2022). For alpha diversity measures we used (i) rarefied richness and (ii) Shannon entropy. Ordination of the ASV table in reduced space (beta diversity) was done using principal coordinate analysis (PCoA). We used two different distance measures in PCoA: (i) Bray–Curtis distance on the ASV abundance table and (ii) hierarchical meta-storms (HMS) (Zhang et al., 2021), a recent functional beta diversity distance. Additionally, the Vegan package was used to perform PERMANOVA analysis to see if the microbial or functional community structures can be explained by different sources of variability. We additionally employed unweighted and weighted UniFrac distances in the PERMANOVA using the Phyloseq package (McMurdie and Holmes, 2013).

To identify the core microbiome, we used the approach discussed in Shade and Stopnisek (2019). The model incorporates a time-specific occupancy model (four different time points for each temperature: *Low*, *Meso*, and *Thermo*). The approach first ranks the ASVs by obtaining a score based on time specific occupancy as well as replication consistency across these temperatures. After ranking the ASVs, Bray–Curtis similarity was calculated between the samples using all ASVs. The subset of core taxa was then constructed by taking the highly ranked ASVs as core and adding an ASV incrementally to this subset, calculating the Bray–Curtis similarity for the subset, and calculating its contribution using C = 1 – BC (subset)/BC(all). The approach stops when addition of an ASV did not cause more than a 2% increase in the explanatory value by Bray–Curtis distance. Independently, a neutral model (Burns et al., 2016) was fitted to the S-shaped abundance-occupancy distributions to inform the ASVs that are likely selected by the environment. These were obtained as those that fall outside the 95% confidence interval of the fitted model and were inferred to be deterministically assembled rather than neutrally selected, with those that are above the model selected by the host environment (red); those points below the model were dispersal limited (blue).

To identify taxa that changed significantly in abundance between different groups, we used the differential heat trees approach (Foster et al., 2017) which highlights differential features on the recovered taxonomy. Additionally, we used the DESeq2 package (Love et al., 2014) to find ASVs that had at least a two log fold difference in abundance between multiple conditions.

Next, to observe the relationship between temperature and the minimal subset of microbes/functions that can explain these parameters, we used the CODA-LASSO approach (Lu et al., 2019) where the abundance of individual covariate $y_{i}$ (temperature in this case) was modeled as regression $y_{i}=\beta_{0}+\beta_{1}\log\left(x_{1i}\right)+\ldots +\beta_{j}\log\left(x_{ji}\right)+\epsilon_{i}$ (for i ^th^ sample and j ^th^ feature, where $x_{ji}$ represents either the microbe or pathway abundance). For this purpose, we used R’s coda4microbiome package (Calle and Susin, 2022). We used the top-100 most abundant genera in the CODA-LASSO model.

## Results and discussion

### Temperature drives diversity shifts

**FIGURE 2:**
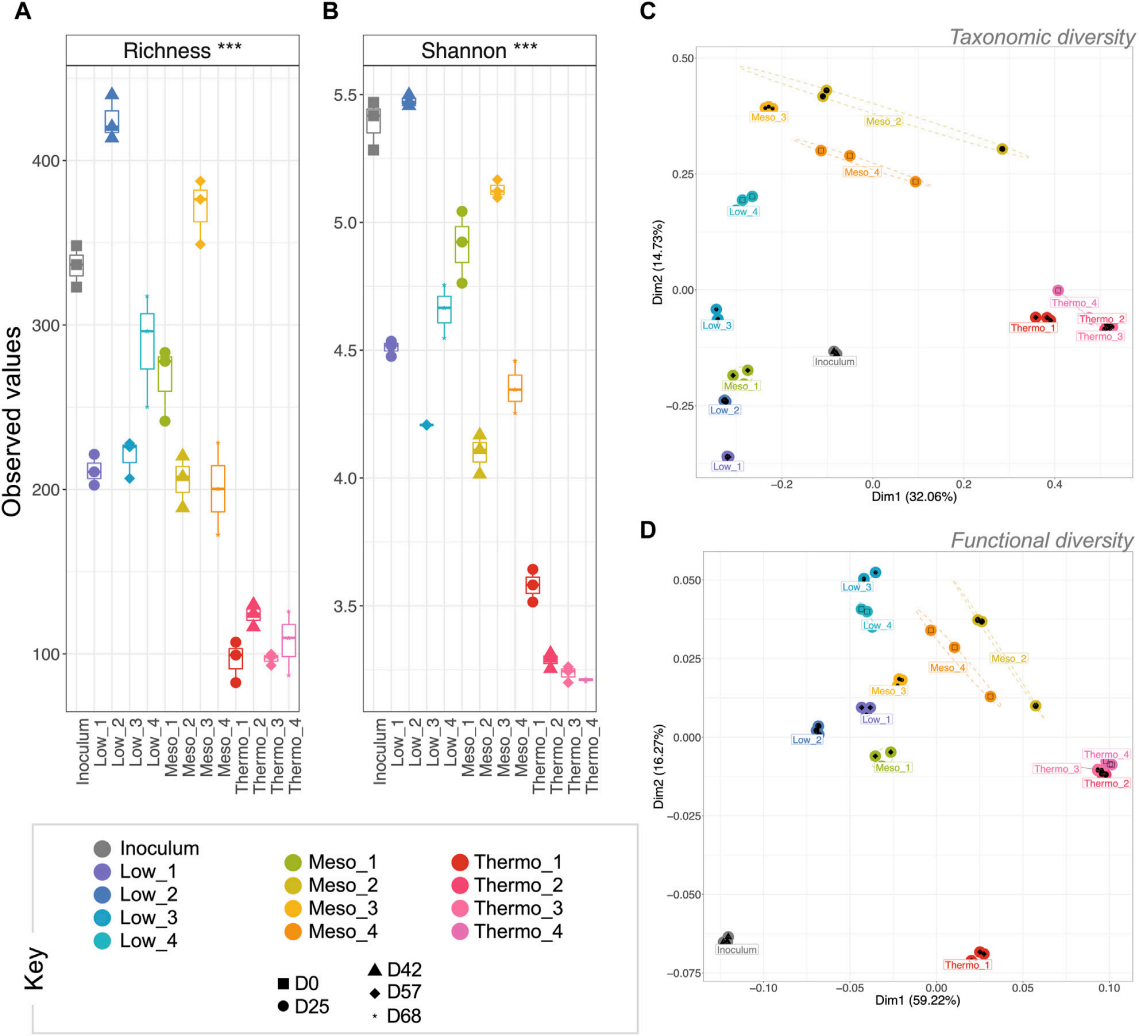
Trajectory of microbial community diversity during a 68-day enrichment trial (DNA-based analysis). Alpha diversity measures are visualized by boxplots of **(A)** rarefied richness and **(B)** Shannon entropy. Beta diversity was measured using **(C)** Bray–Curtis distances for taxonomic beta diversity and **(D)** hierarchical meta-storm for functional beta diversity. Both are visualized on principal coordinate analysis (PCoA) plotting. Samples are indicated by color, and the ellipses for **(C)** are drawn at a 95% CI for all samples from each category. Significant differences for **(A,B)** (ANOVA) indicated as * (*p* < 0.05), ** (*p* < 0.01), or *** (*p* < 0.001).

The selective pressure of the three applied temperatures had a pronounced effect on microbial community diversity and community profile. In particular, the rarefied richness (Figure 2) and Shannon entropy (Figure 2) at thermophilic temperatures was significantly more reduced (*p* < 0.001 for both) than the low and mesophilic communities. This suggests that the thermophilic community was increasingly dominated by a single or a sub-group of taxa, while the low and mesophilic communities remained comparatively more balanced in terms of abundance; this is a well-documented phenomenon with respect to thermophilic diversity. Reduced diversity/richness at increased temperatures has been observed in both natural hot springs (Cole et al., 2013; Podar et al., 2020), geothermal soil fields (Li et al., 2015), geothermal steam vents (Benson et al., 2011), and engineered anaerobic digestion systems (Gagliano et al., 2015; Moset et al., 2015; Zhu et al., 2017; Ao et al., 2021). To our knowledge, this is the first record of thermophilic PHA production, but the diversity trends do appear to mirror other natural and engineered systems.

Although the microbiomes continued to develop over the course of the enrichment phase, analysis of the taxonomic beta diversity revealed a significant change between the inoculum and the first time-point (e.g., “Low_1”) at each temperature (Figure 2). This suggested that the imposed reactor conditions (e.g., temperature, pH, feast-famine feeding regime, etc.) all resulted in a major shift in the community structure from the original inoculum. These reactor conditions produced similar initial effects on the low and mesophilic communities (close distances between “Low_1” and “Meso_1”; Figure 2), which both continued to develop throughout the phase. Conversely, the thermophilic temperatures quickly resulted in a significantly different community structure with only minimal changes thereafter.

PERMANOVA suggested that temperature was the strongest driver of community structure for every beta diversity distance metric (*p* = 0.001), explaining nearly 50% of variation between samples (Table 1). Indeed, except for the ammonium concentration entering the system—which was constant across the reactors—every other environmental co-variate revealed a significant effect on microbial community diversity (Table 1). In particular, the pH, VS concentration and effluent ammonium concentrations all significantly correlated with the changes in community structure. It was previously reported for this experiment that ammonium uptake occurred during the feast phase and that higher temperatures resulted in faster uptake rates (Palmeiro-Sánchez et al., 2023). It is therefore unsurprising that the ammonium effluent concentrations at each temperature were significantly correlating with diversity parameters. It is also worth noting that pH may also have played a significant role in shaping the community—a common phenomenon in environmental microbiology (Högberg et al., 2007; Meron et al., 2011; Liu et al., 2018; Trego et al., 2020a). The pH values were reported as 8.6 (low), 8.9 (mesophilic), and 9.1 (thermophilic) (Palmeiro-Sánchez et al., 2023). Although the community was significantly correlated with the variations in pH, none of these reactors in reality was operating at an optimal, neutral pH range (Villano et al., 2010). The alkaline conditions were likely a selective pressure for all the reactors. Indeed, in other wastewater treatment systems operating at alkaline pH, similar community profiles were observed. *Paracoccus*, *Alishewanella*, and *Pseudomonas* in particular can tolerate higher pH levels (Yang et al., 2011). This could have several process implications, and there may be future opportunities to assess PHA-accumulating communities under more neutral conditions.

**TABLE 1:** PERMANOVA values between environmental co-variates measured on the day of biomass sampling and beta diversity distances from DNA-based microbial community analysis of the 16S rRNA gene. Significant differences are represented as * (*p* < 0.05), ** (*p* < 0.01), or *** (*p* < 0.001).

Covariate	Bray–Curtis	Unweighted UniFrac	Weighted UniFrac	Functional hierarchical meta-storm
Groups	*R* ^2^ = 0.901 (*p* = 0.001***)	*R* ^2^ = 0.537 (*p* = 0.001***)	*R* ^2^ = 0.904 (*p* = 0.001***)	*R* ^2^ = 0.987 (*p* = 0.001***)
Temp	*R* ^2^ = 0.497 (*p* = 0.001***)	*R* ^2^ = 0.220 (*p* = 0.001***)	*R* ^2^ = 0.459 (*p* = 0.001***)	*R* ^2^ = 0.687 (*p* = 0.001***)
pH	*R* ^2^ = 0.137 (*p* = 0.001***)	*R* ^2^ = 0.084 (*p* = 0.001***)	*R* ^2^ = 0.203 (*p* = 0.001***)	*R* ^2^ = 0.303 (*p* = 0.001***)
sCOD Out	*R* ^2^ = 0.153 (*p* = 0.002**)	*R* ^2^ = 0.072 (*p* = 0.001***)	*R* ^2^ = 0.250 (*p* = 0.001***)	*R* ^2^ = 0.144 (*p* = 0.003**)
TS	*R* ^2^ = 0.078 (*p* = 0.02*)	*R* ^2^ = 0.051 (*p* = 0.001***)	*R* ^2^ = 0.051 (*p* = 0.143)	*R* ^2^ = 0.067 (*p* = 0.098)
VS	*R* ^2^ = 0.110 (*p* = 0.004**)	*R* ^2^ = 0.068 (*p* = 0.001***)	*R* ^2^ = 0.132 (*p* = 0.001***)	*R* ^2^ = 0.244 (*p* = 0.001***)
VS/TS	*R* ^2^ = 0.316 (*p* = 0.001***)	*R* ^2^ = 0.109 (*p* = 0.001***)	*R* ^2^ = 0.242 (*p* = 0.001***)	*R* ^2^ = 0.507 (*p* = 0.001***)
Ammonium in	N.S.	N.S.	N.S.	N.S.
Ammonium out	*R* ^2^ = 0.244 (*p* = 0.001***)	*R* ^2^ = 0.078 (*p* = 0.001***)	*R* ^2^ = 0.189 (*p* = 0.001***)	*R* ^2^ = 0.319 (*p* = 0.001***)
Feast % of cycle	*R* ^2^ = 0.149 (*p* = 0.005**)	*R* ^2^ = 0.083 (*p* = 0.002**)	*R* ^2^ = 0.118 (*p* = 0.014*)	*R* ^2^ = 0.039 (*p* = 0.002**)
Feast length	*R* ^2^ = 0.149 (*p* = 0.004**)	*R* ^2^ = 0.083 (*p* = 0.003**)	*R* ^2^ = 0.118 (*p* = 0.007**)	*R* ^2^ = 0.217 (*p* = 0.005**)
Sampling day	*R* ^2^ = 0.109 (*p* = 0.002**)	*R* ^2^ = 0.062 (*p* = 0.002**)	*R* ^2^ = 0.118 (*p* = 0.001***)	*R* ^2^ = 0.199 (*p* = 0.002**)

Overall, similar trends were reflected in the functional diversity (Figure 2). Moreover, in terms of functional diversity, a more pronounced gradient from low to high temperature was observed. This suggests greater functional differences between the low and high temperature communities. PERMANOVA again confirmed that temperature was a primary driver of functional diversity, explaining nearly 70% (*p* = 0.001) of the variability between categories (Table 1).

### Effective selection of diverse PHA accumulating consortia

One of the primary objectives of this study was to assess the differences between PHA-accumulating consortia at different temperature ranges and the feasibility of operating PHA production at non-optimal temperatures. Indeed, temperature is an important process parameter in terms of reaction rates, microbial community efficiencies, and overall energy consumption. Efficient operation at lower temperatures could dramatically reduce operational costs, resulting in a more cost-effective process. Alternatively, if reaction rates and yields at higher temperatures are significantly higher, the faster/increased production may offset the cost of heating, making high temperatures more economical.

**FIGURE 3:**
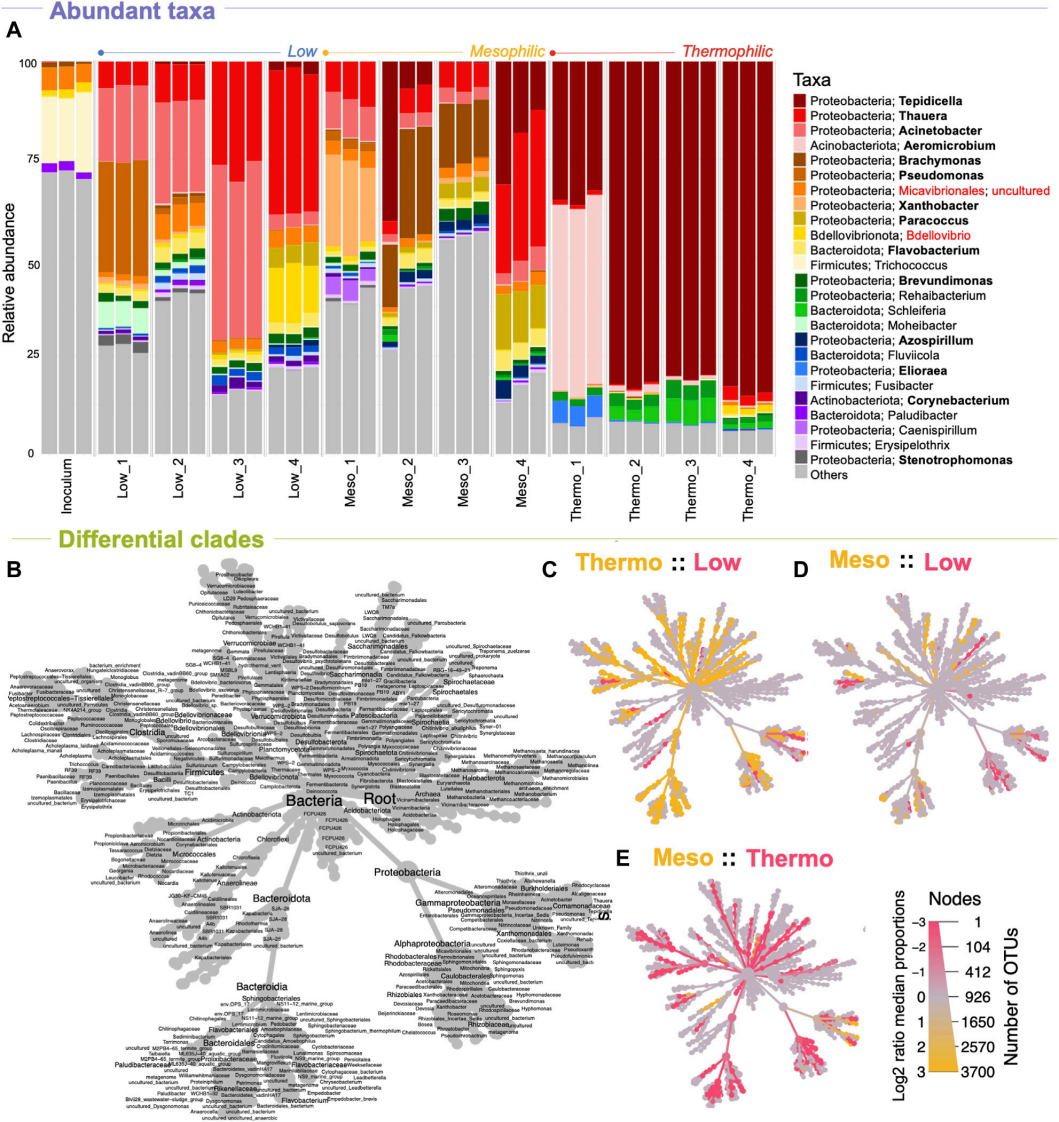
Microbial community structure across temperature and time (DNA-based analysis). The makeup and development of the microbial community at each timepoint shown as **(A)** the relative abundances of the top-25 most abundant genera according to variances in the 16S rRNA genes, where “others” indicate anything that is not in the top 25. Differentially abundant clades between the various temperatures are highlighted in the heat trees **(C–E)** and can be identified using **(B)** the reference tree. Known PHA-storing genera are highlighted in bold; known predators are highlighted in red.

PHA accumulation was previously reported for this experiment at each of the temperature ranges, with mesophilic temperatures outperforming the other two in terms of overall PHA production and production rates (Figure 1) (Palmeiro-Sánchez et al., 2023). Analysis of the microbial community in this study showed that a highly abundant PHA accumulating consortium was indeed achieved at each temperature (Figure 3). Of the top-25 most abundant genera, 13 are documented PHA accumulators. At each temperature range, the most abundant genera (by the final timepoint in the enrichment phase) were PHA accumulators. At low temperatures, these were *Thauera*, *Acinetobacter*, and *Paracoccus*. At mesophilic temperatures these were *Tepidicella*, *Thauera*, *Acinetobacter*, *Paracoccus*, *Flavobacterium*, and *Azospirillum*. Finally, at thermophilic temperatures, the abundant PHA accumulators were primarily *Tepidicella* (>80% relative abundance) and *Thauera*.

Notably, as predicted by the diversity analysis, the thermophilic system was heavily dominated by *Tepidicella*, a genus originally proposed in 2006 with a novel species isolated from a geothermal area in the Azores (França et al., 2006). Although PHA accumulation was unexamined in 2006, a second novel species was added to the genus in 2019, *T. baoligensis*, which was reported to accumulate polyhydroxybutyrate (PHB) (You et al., 2019). Thence, PHA biosynthesis has been identified in both species at the genotype level. Indeed, a wide range of thermophilic bacteria have since been reported to store PHA (Obruča et al., 2022). Although other thermophilic PHA accumulators were present, our system was dominated by *Tepidicella*, which appeared to out-compete all other substrate competitors.

Next, heat-tree analysis corroborated diversity trends, showing that low and mesophilic microbiomes display only minimal differences in community structure, with significant differences often only occurring at higher-level taxonomic ranks (family/genus level; Figure 3). Thermophilic communities, however, showed many significant differences at lower-level taxonomic ranks compared to both low and mesophilic communities (Figure 3). These included the enrichment of clades within *Desulfobacterota*, *Firmicutes*, *Bacteroidota*, and several unique *Proteobacteria*, which were all significantly less abundant at reduced temperatures.

Overall, the microbiome analysis revealed that an enriched, highly abundant PHA-accumulating consortium was achieved at each temperature. Moreover, at each temperature, the structure of the PHA-accumulating consortium was unique, with the most extreme differences at thermophilic temperatures. Notably, while the PHA-accumulating consortium was selected by the reactor conditions, none of these organisms were highly abundant in the starting inoculum. Instead, they were successfully enriched throughout the enrichment phase. Finally, the most diverse PHA-accumulating community was observed at mesophilic temperatures, which could help explain the optimal process performance from this temperature range.

#### Thauera: synthesizing PHA irrespective of temperature

Identifying the core microbiome is a useful means of determining which organisms are persistent across a set of samples/conditions. Although the core microbiome has been defined in many ways throughout the literature (Trego et al., 2021a), we here incorporated a time-specific occupancy model (four different time points for each temperature—*Low*, *Meso*, and *Thermo*). The approach first ranks the ASVs by obtaining a score based on time specific occupancy as well as replicate consistency across these temperatures (more details in “Materials and Methods”).

**FIGURE 4:**
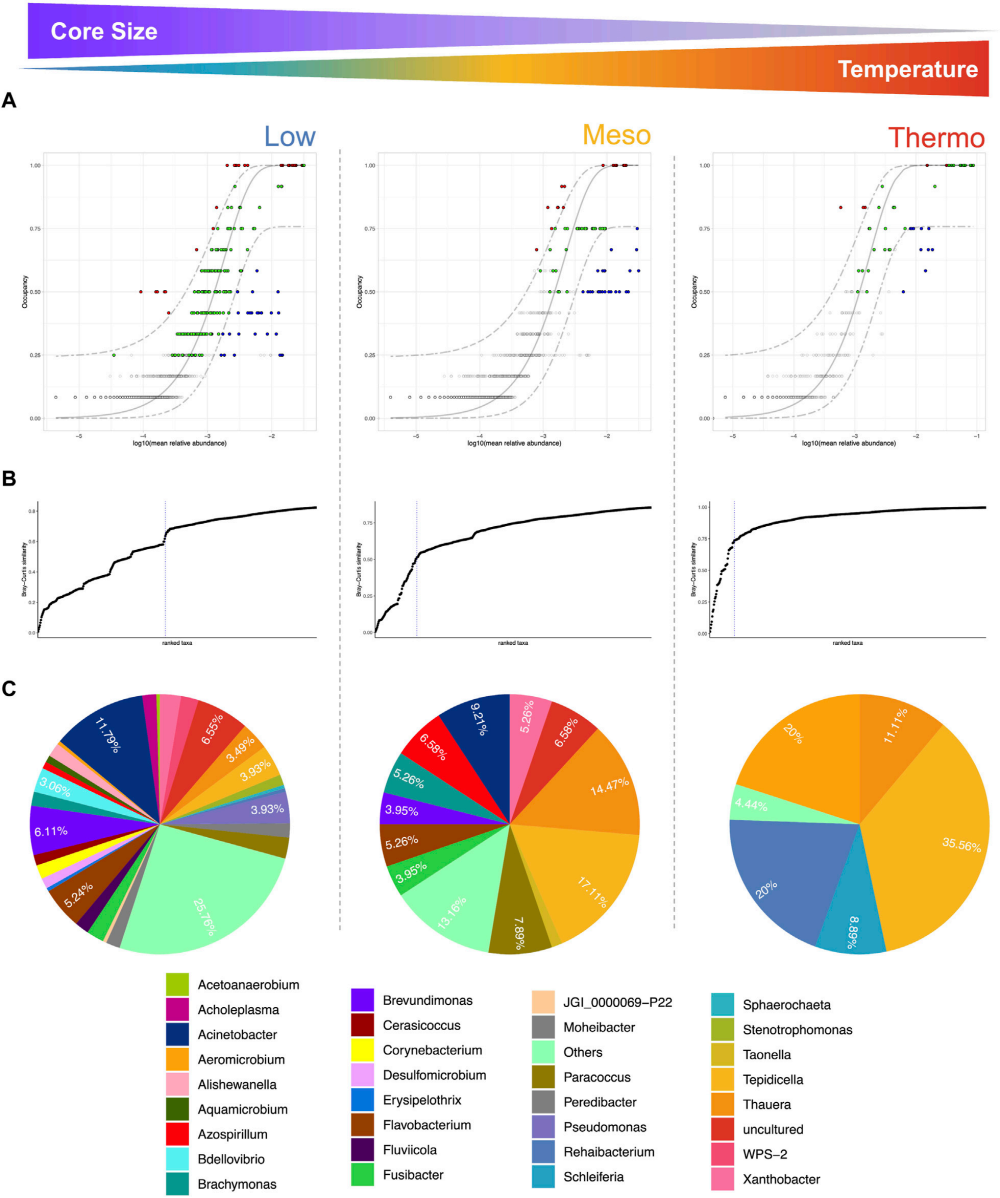
Core microbiome at the three temperatures (DNA-based analysis). The core was identified using **(A)** species occupancy abundance diagrams. These incorporated (for each temperature) a time-specific occupancy model (time being the four time-points sampled during Phase 1). Once ASV rankings were obtained based on ASV occupancy within these compartments and their replicate consistency, Bray–Curtis similarity was calculated for the whole dataset and also for only the top-ranked taxa. The contribution of the top-ranked taxa was divided by the total Bray–Curtis similarity to calculate a percentage contribution of the prospective core set to beta diversity. In **(B),** the next ranked taxon was added consecutively to find the point in the ranking at which adding one more taxon offered diminishing returns on explanatory value for beta diversity. The blue dotted line represents the “last 2% decrease” criteria where ASVs are incorporated in the core subset until there is no more than a 2% decrease in beta diversity. Those ASVs that are identified as part of the core microbiome are colored either red, green, or blue in **(A)**. Independently, a neutral model was fitted and those ASVs that fall within the 95% confidence interval were colored green. Non-neutral ASVs that fall above the model were selected by the host environment, while those below the neutral model were assembled via dispersal limitation. Finally, pie charts in **(C)** represent the genus-level assignments of the core ASVs.

**FIGURE 5:**
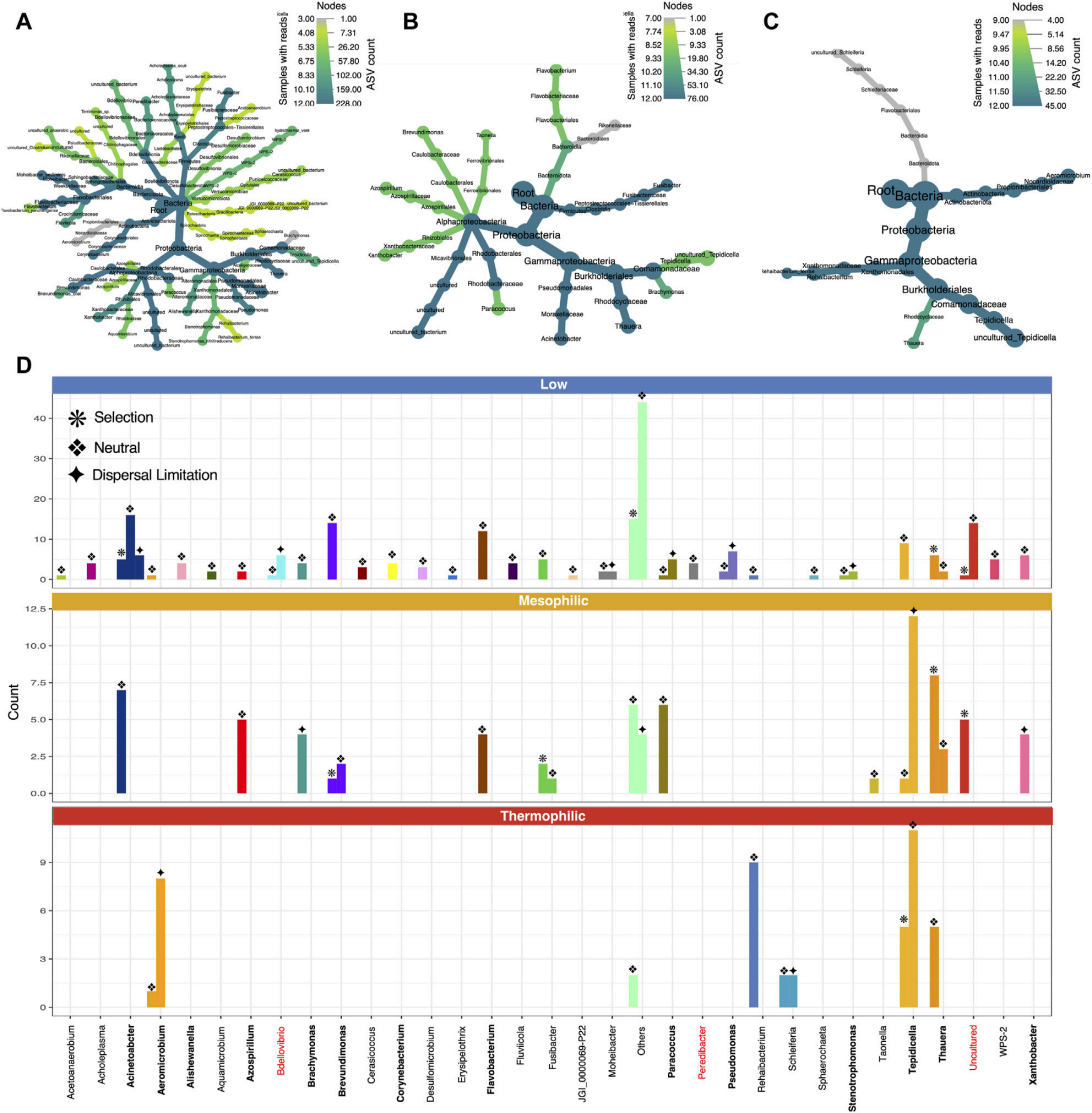
Taxonomic coverage and assembly mechanisms of the core microbiome (DNA-based analysis). Heat tree representation of core microbiome at **(A)** low **(B)** mesophilic and **(C)** thermophilic temperatures; and **(D)** bar plot of neutral/non-neutral ASVs at genus level. Known PHA accumulators are highlighted in bold, while predatory groups are highlighted in red.

Each temperature range yielded a unique core microbiome, with some species overlap (Figure 4), particularly in terms of the Proteobacteria, where we find PHA-accumulating organisms (Figure 5). The size of the core microbiome decreased with increasing temperature so that the core of the thermophilic microbiome only consisted of five genera (Figures 4, 5). *Thauera* and *Tepidicella*, both known PHA-accumulating organisms (You et al., 2019; Colpa et al., 2020; Andreolli et al., 2022), were the only taxa present in the core at each temperature, although *Tepidicella* was in very low abundance at low temperatures. *Thauera* has been repeatedly reported to dominate during mixed culture PHA production (Sruamsiri et al., 2020; Tamang et al., 2021; Huang et al., 2022) using a wide variety of substrates (Jiang et al., 2011b; Carvalho et al., 2014; Coats et al., 2016; Clagnan and Adani, 2023) and also at high salinity (Wen et al., 2024). With the exception of several studies which were operated at ambient temperatures (∼20–25 °C) (Carvalho et al., 2014; Coats et al., 2016; Sruamsiri et al., 2020), most studies report mixed culture PHA production at mesophilic temperatures and rarely at low (<20°C) or high temperatures. This study is the first instance where *Thauera* and *Tepidicella* have been shown to be among the core microbiome at temperatures between 15 °C and 48 °C. This suggests that these genera are highly cosmopolitan with respect to temperature.

Moreover, the presence of several predatory genera—*Micavibrionales*, *Bdellovibrio*, and *Peredebacter*—also indicates the enrichment of a whole network of inter-related species (Davidov and Jurkevitch, 2004; Sockett, 2009; Dashiff et al., 2011; Bratanis et al., 2020). These genera all belong to a category of predatory bacteria called *Bdellovibrio*-and-like-organisms (BALOs), and the predatory mechanism has been described in detail for *Bdellovibrio* (Davidov and Jurkevitch, 2004; Bratanis et al., 2020). These obligate predators are known to parasitize other Gram-negative bacteria by invading their periplasmic space. This is accomplished by creating a pore in the outer membrane and crossing via the peptidoglycan layer (Bratanis et al., 2020; Gonzalez et al., 2021). It enters the growth phase inside its host, where it hydrolyzes and consumes complex biopolymers within the host, including some types of host-synthesized PHA (Wijeyekoon et al., 2018). Indeed, these predators have even recently been suggested as a mechanism for PHA recovery, although research into this is in its infancy (Martínez et al., 2016; Gonzalez et al., 2021). Although BALOs have been reported in several mixed-culture PHA synthesizing systems, in most cases they do not appear to have a dramatic effect on PHA yields or abundances of PHA-synthesizing organisms (Wijeyekoon et al., 2018; Crognale et al., 2019; Correa-Galeote et al., 2022), which also appeared to be the case in our systems.

#### Temperature influences microbiome assembly and structure

**FIGURE 6:**
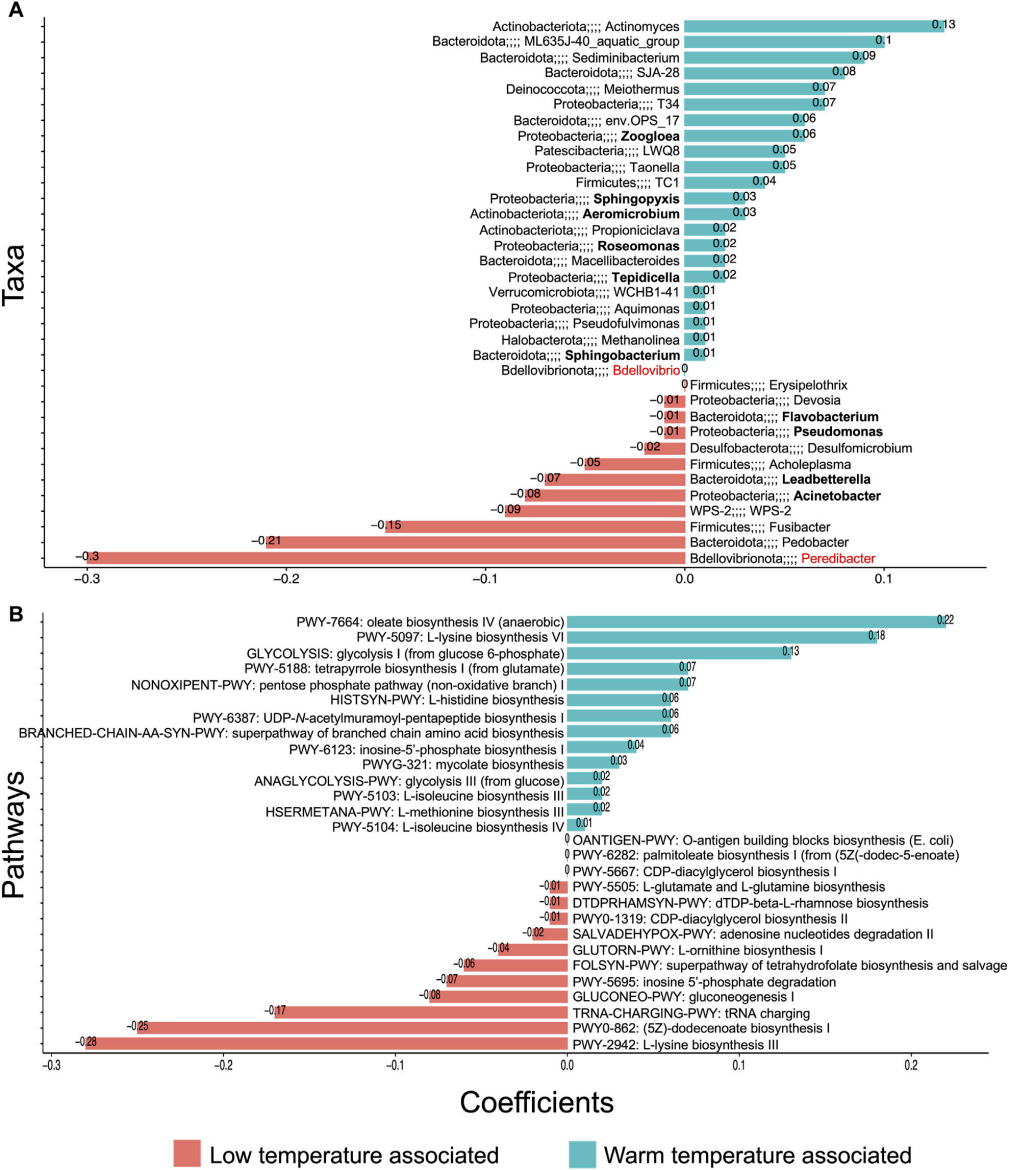
Significant relationships between microbial community and temperature (DNA-based analysis). Temperature-associated **(A)** genera and **(B)** pathways where **(A)** and **(B)** both represent the ß-coefficients returned from the CODA-LASSO procedure as two disjoint sets—those that are positively related (blue) and those that are negatively related (red) to temperature increases.

Although some PHA-accumulating organisms were found to be abundant and core irrespective of temperature (e.g., *Thauera*), others were more temperature-sensitive. In particular, *Zoogloea*, *Sphingopyxis*, *Aeromicrobium*, and *Roseomonas* were positively associated with warmer temperatures (Figure 6). Meanwhile, *Leadbetterella* and *Acinetobacter* were associated with lower temperatures. Indeed, especially in terms of the core microbiome, low temperatures harbored a greater number of genera (Figures 4, 5). Moreover, while most core groups fit the neutral model of microbial community assembly, changes in temperature did appear to shift the assembly mechanisms for some genera. In particular, *Brachymonas*, *Aeromicrobium, Tepidicella*, and *Thauera* all observed shifts in mode of assembly. *Thauera*, for example, fit the neutral model at high temperatures, where its growth temperature is optimal (Heider and Fuchs, 2015), but at mesophilic and low temperatures it was assembled more frequently via selective processes (Figure 5).

Additionally, we assessed which functions (at pathway level) were associated with temperature (Figure 6). It was observed that while several amino acid and fatty acid biosynthesis pathways were temperature-associated, storage compound biosynthesis pathways (to which PHA accumulation belongs) were not identified as temperature-dependent. This suggests that at each temperature, a sufficiently abundant PHA accumulating consortium was established.

#### The active community is dominated by PHA accumulating organisms

Following the enrichment phase, where the feast–famine feeding regime and temperature were selecting for PHA accumulating organisms, cDNA was sequenced to provide insights into the active fraction of the microbiome during the enrichment and accumulation phases. DNA shows us the profile or structure of the total community containing dead or dormant cells, while the cDNA yields a profile of the active fraction of the microbial community (De Vrieze et al., 2016). Several studies have used cDNA for wastewater treatment (Cerrillo et al., 2016; De Vrieze et al., 2018; Trego et al., 2021b). The efficiency of these processes relies on collaboration between micro-organisms, and this collaboration is not accurately represented by DNA-based studies (De Vrieze et al., 2018). Therefore, cDNA was sequenced for these samples so that the active and functioning community members producing PHA could be identified.

During the accumulation phase, the reactors were pulse-fed. As soon as changes in the dissolved oxygen readings suggested that the substrate had been completely utilized, more feed was supplied. This constant supply of substrate is known to stimulate PHA accumulation (Palmeiro-Sánchez et al., 2023). We chose to sequence cDNA during this phase in order to compare the active fraction of the microbial community at the three different temperatures. In this way, we hoped to highlight the proportion of the community that was actively participating in PHA accumulation.

**FIGURE 7:**
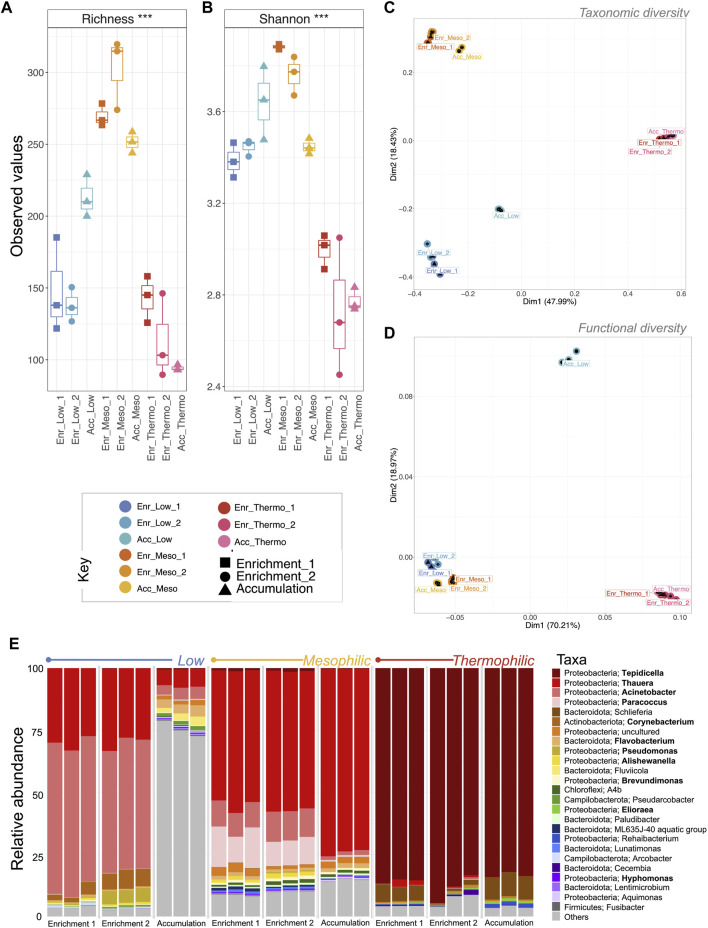
Changes in the active fraction of microbial community diversity and abundance during the enrichment and accumulation phases of PHA production (cDNA-based analysis). Alpha diversity measures are visualized by boxplots of **(A)** rarefied richness and **(B)** Shannon entropy. Beta diversity was measured using **(C)** Bray–Curtis distances for taxonomic beta diversity and **(D)** hierarchical meta-storm for functional beta diversity. Both were visualized on a PCoA plot where samples are indicated by color, and ellipses are drawn at a 95% CI for all samples from each category. Significant differences for **(A)** and **(B)** (ANOVA) indicated as * (*p* < 0.05), ** (*p* < 0.01), or *** (*p* < 0.001). Finally, the community structure was visualized as **(E)** relative abundances of the top 25 most abundant genera according to variances in the 16S rRNA genes, where known PHA-storing genera are highlighted in bold and “others” indicate anything that is not in the top 25.

In terms of the active fraction of the microbiome, the mesophilic systems had the highest richness and Shannon entropy (Figure 7) compared to the higher and lower temperatures. Taxonomic beta diversity showed that, similar to DNA analysis, temperature again played a significant role in shaping the structure of the microbiome (Figure 7; Table 2; PERMANOVA: *p* = 0.001). Additionally, the microbial community abundances were significantly correlated with recovered PHA concentrations (Table 2; PERMANOVA: *p* = 0.008). In terms of functional diversity, the low and mesophilic systems clustered tightly while the thermophilic samples clustered apart (Figure 7). Interestingly, temperature was the only environmental co-variate, which strongly correlated with functional diversity (Table 2).

**TABLE 2:** PERMANOVA values between environmental co-variates measured on the day of sampling and beta diversity distances from cDNA-based microbial community analysis of the 16S rRNA gene. Significant differences are represented as * (*p* < 0.05), ** (*p* < 0.01), or *** (*p* < 0.001).

Covariate	Bray–Curtis	Unweighted UniFrac	Weighted UniFrac	Functional hierarchical meta-storm
Groups	*R* ^2^ = 0.978 (*p* = 0.001***)	*R* ^2^ = 0.635 (*p* = 0.001***)	*R* ^2^ = 0.982 (*p* = 0.001***)	*R* ^2^ = 0.995 (*p* = 0.001***)
Temp	*R* ^2^ = 0.651 (*p* = 0.001***)	*R* ^2^ = 0.110 (*p* = 0.037*)	*R* ^2^ = 0.626 (*p* = 0.001***)	*R* ^2^ = 0.727 (*p* = 0.001***)
Stage	*R* ^2^ = 0.125 (*p* = 0.108)	*R* ^2^ = 0.727 (*p* = 0.001***)	*R* ^2^ = 0.137 (*p* = 0.084)	*R* ^2^ = 0.087 (*p* = 0.296)
OLR	*R* ^2^ = 0.122 (*p* = 0.019*)	*R* ^2^ = 0.076 (*p* = 0.006**)	*R* ^2^ = 0.077 (*p* = 0.1)	*R* ^2^ = 0.054 (*p* = 0.244)
PHA by TS weight %	*R* ^2^ = 0.125 (*p* = 0.008**)	*R* ^2^ = 0.076 (*p* = 0.009**)	*R* ^2^ = 0.071 (*p* = 0.124)	*R* ^2^ = 0.063 (*p* = 0.175)
PHA g/L	*R* ^2^ = 0.109 (*p* = 0.028*)	*R* ^2^ = 0.072 (*p* = 0.012*)	*R* ^2^ = 0.060 (*p* = 0.181)	*R* ^2^ = 0.052 (*p* = 0.294)

At each temperature, the active populations were dominated by PHA-accumulating organisms. At low temperatures, these were primarily *Thauera*, *Acinetobacter*, *Corynebacterium*, and *Pseudomonas*. At mesophilic temperatures, *Thauera*, *Acinetobacter*, and *Paracoccus* were the dominant genera, while at thermophilic temperatures the community was heavily dominated by *Tepidicella* (∼85% relative abundance).

#### Accumulation dosing strategy unsuccessful at low temperatures

**FIGURE 8:**
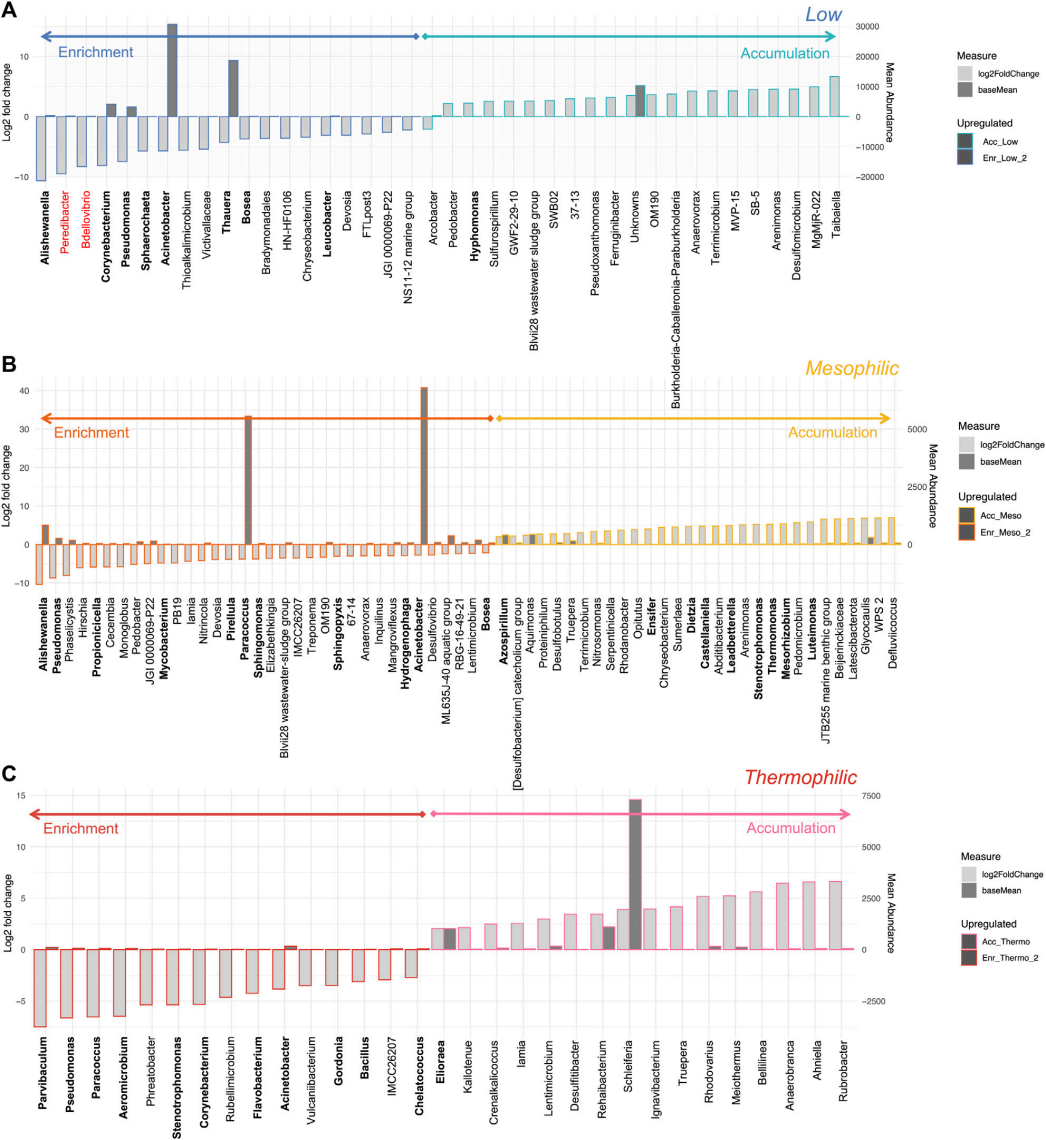
Differential abundance analysis of key genera between the enrichment and accumulation phases of operation (cDNA analysis). The bar charts identify any genera with a Log2 fold change in abundance between groups (*y*-axis on the left and light gray bar) and the mean abundance across all the samples (*y*-axis on the right and dark gray bar) for the **(A)** low, **(B)** mesophilic, and **(C)** thermophilic systems. Genera in greater abundance in the enrichment phase are shown with a darker border on the left side of the chart; genera which were more abundant in the accumulation phase are shown on the right with a lighter border. Any taxa which are known PHA accumulators are highlighted in bold, while those in red font are known predators of PHA-accumulating organisms.

For the mesophilic and thermophilic systems, minimal changes in the active fraction of the community structure were observed between the enrichment and accumulation phases (Figure 7). This, however, was not the case for the low temperature system, where the change in feeding regime, designed to stimulate PHA accumulation, resulted in a collapse of the enriched microbial community. This is evident in terms of the taxonomic and functional diversity, as well as the loss of abundant PHA-accumulating organisms. It was reported that the mesophilic system out-performed the low and thermophilic systems in terms of total PHA concentrations recovered and with respect to recovery rates (Palmeiro-Sánchez et al., 2023). Analysis of the differential taxa between the enrichment and accumulation phases at each temperature revealed an interesting pattern (Figure 8). For both the low and thermophilic systems, the enrichment phase contained many PHA accumulating organisms that became significantly reduced in relative abundance during the accumulation phase. This was not observed for the mesophilic system, which saw several of the PHA accumulating organisms increase in relative abundance.

It would seem that the dosing strategy (in which carbon was added in pulses whenever the dissolved oxygen increased in the liquid media), which worked well at the mesophilic range, was particularly ineffective at low temperatures. Future work would be required to determine whether the feeding during this phase was too frequent or not frequent enough. Given that the enrichment communities at low temperatures were diverse and contained an active PHA-accumulating consortium, it is plausible that the low temperature process could be feasible under the right conditions. Indeed, optimization of the accumulation phase at low temperatures could have major implications for net energy consumption. This is perhaps an interesting case where a view of the microbial community can help guide data-driven process optimization.

## Conclusion

We here report on the community dynamics of mixed-culture PHA production at three temperatures: low, mesophilic, and thermophilic. The feast–famine feeding regime provided sufficient selective pressure to enrich for a diverse PHA-accumulating microbial consortium at each temperature. Community structure was temperature-dependent, and most taxa were temperature-sensitive. The active fraction of the microbial community was particularly dominated by PHA-accumulating microorganisms. At low temperatures, these were primarily *Thauera*, *Acinetobacter*, *Corynebacterium*, and *Pseudomonas*. At mesophilic temperatures, *Thauera*, *Acinetobacter*, and *Paracoccus* were the dominant genera, while at thermophilic temperatures the community was heavily dominated by *Tepidicella*. *Thauera*, however, was abundant irrespective of temperature. The dosing strategy (pulse feeding) applied during the accumulation phase worked for the mesophilic and thermophilic systems but resulted in community collapse at low temperatures, leading to low PHA yields. Further optimizations at low temperatures, however, could resolve this issue and result in a more efficient low-temperature process.

## Data Availability

The datasets presented in this study can be found in online repositories. The names of the repository/repositories and accession number(s) can be found at https://www.ncbi.nlm.nih.gov/, PRJNA907847.
